# The autophagy marker LC3 strongly predicts immediate mortality after surgical resection for hepatocellular carcinoma

**DOI:** 10.18632/oncotarget.19763

**Published:** 2017-08-01

**Authors:** Chih-Wen Lin, Chih-Che Lin, Po-Huang Lee, Gin-Ho Lo, Pei-Min Hsieh, Kah Wee Koh, Chih-Yuan Lee, Yao-Li Chen, Chia-Yen Dai, Jee-Fu Huang, Wang-Long Chuang, Yaw-Sen Chen, Ming-Lung Yu

**Affiliations:** ^1^ Division of Gastroenterology and Hepatology, E-Da Dachang Hospital, I-Shou University, Kaohsiung, Taiwan; ^2^ Division of Gastroenterology and Hepatology, Department of Medicine, E-Da Hospital, I-Shou University, Kaohsiung, Taiwan; ^3^ Health Examination Center, E-Da Hospital, I-Shou University, Kaohsiung, Taiwan; ^4^ School of Medicine, College of Medicine, I-Shou University, Kaohsiung, Taiwan; ^5^ Department of Surgery, E-Da Hospital, I-Shou University, Kaohsiung, Taiwan; ^6^ Department of Surgery, Kaohsiung Chang Gung Memorial Hospital and Chang Gung University College of Medicine, Kaohsiung, Taiwan; ^7^ Department of Surgery, National Taiwan University Hospital, Taipei, Taiwan; ^8^ Department of Surgery, Changhua Christian Hospital, Changhua, Taiwan; ^9^ Hepatobiliary Division, Department of Internal Medicine and Hepatitis Center, Kaohsiung Medical University Hospital and Center for Infectious Disease and Cancer Research, Kaohsiung Medical University, Kaohsiung, Taiwan; ^10^ Institute of Biomedical Sciences, National Sun Yat-sen University, Kaohsiung, Taiwan; ^11^ Liver Center, Division of Gastroenterology, Massachusetts General Hospital, Harvard Medical School, Boston, MA, USA

**Keywords:** albumin, autophagy, liver failure, post-hepatectomy immediate mortality, predictor

## Abstract

The remnant liver's ability to regenerate may affect post-hepatectomy immediate mortality. The promotion of autophagy post-hepatectomy could enhance liver regeneration and reduce mortality. This study aimed to identify predictive factors of immediate mortality after surgical resection for hepatocellular carcinoma (HCC). A total of 535 consecutive HCC patients who had undergone their first surgical resection in Taiwan were enrolled between 2010 and 2014. Clinicopathological data and immediate mortality, defined as all cause-mortality within three months after surgery, were analyzed. The expression of autophagy proteins (LC3, Beclin-1, and p62) in adjacent non-tumor tissues was scored by immunohistochemical staining. Approximately 5% of patients had immediate mortality after surgery. The absence of LC3, hypoalbuminemia (<3.5 g/dl), high alanine aminotransferase, and major liver surgery were significantly associated with immediate mortality in univariate analyses. Multivariate logistic regression demonstrated that absence of LC3 (hazard ratio/95% confidence interval: 40.8/5.14-325) and hypoalbuminemia (2.88/1.11-7.52) were significantly associated with immediate mortality. The 3-month cumulative incidence of mortality was 12.1%, 13.0%, 21.4% and 0.4%, respectively, among patients with absence of LC3 expression, hypoalbuminemia, both, or neither of the two. In conclusion, the absence of LC3 expression in adjacent non-tumor tissues and hypoalbuminemia were strongly predictive of immediate mortality after resection for HCC.

## INTRODUCTION

Patients with liver cirrhosis are at risk of developing hepatocellular carcinoma (HCC), and the severity of cirrhosis has an impact on post-hepatectomy-associated morbidity and mortality [1]. Liver resection (LR) is often considered the standard management for resectable HCC, and the severity of cirrhosis based on histological examination and the evaluation of hepatic functional reserve have been used as guidelines to distinguish patients suitable for curative LR [1–3]. With the improvement in techniques for the early detection of HCC, perioperative management and operative techniques in the last three decades, surgical resection has become a more favorable option for HCC patients [4–6]. Unfortunately, LR is one of the most complex operative therapies, and the risks associated with post-hepatectomy complications are relatively high [7]. Extended LR in patients with cirrhotic liver and staged LR are being applied as a means for curative resection and enhancement of long-term survival. This compromises liver function and the small functional remnant liver volumes in these patients increases the risk of developing post-hepatectomy liver failure (PHLF) and mortality [8]. The mortality rate has been reported to be as high as 30% after major LR, with PHLF being the predominant cause of morbidity and mortality [7–9]. Although great improvement in outcomes after major LR has been attributed to the improved in operative management and advances in post-operative critical care, a mortality rate of up to 10% is still being observed [10, 11]. Thus, PHLF and mortality remain a critical issue. Several studies have reported that factors including the patient's conditions, surgical management, and post-operative assessment may be used to predict PHLF and mortality [7, 8, 12–16]. However, these factors are not consistent in predicting immediate mortality (IM), which is defined as death, regardless of cause, occurring within 3 months following LR. The identification of predictive factors of IM after surgical resection is of major clinical relevance and may serve as a promising strategy to decrease mortality among HCC patients.

Autophagy is a process through which damaged organelles are delivered to the lysosome for degradation, recycling, and energy generation [17–20]. It plays an important role in the physiology and pathogenesis of human liver diseases [18, 20, 21]. Recently, autophagy markers (LC3 or Beclin-1) have been reported to show controversial results in the prognosis of overall survival in HCC patients after hepatectomy [22–25]. In a mouse model, the suppression of autophagy impairs hepatocyte senescence and reduces energy provision required for liver regeneration [26]. Similarly, mice with a partial hepatectomy exhibit induced autophagy, and treatment with autophagy-inducing amiodarone is significantly associated with an increase in liver growth and regeneration, accompanied by a reduction in liver injury [27]. These studies suggest that autophagy has an impact on liver regeneration after partial hepatectomy. This raises the need to investigate the potential relationship between autophagy and post-hepatectomy IM in humans. In the current study, we aimed to investigate the role of hepatic autophagy marker(s) present in the adjacent non-tumor (ANT) tissues in predicting mortality within three months after LR for HCC. We hypothesized that autophagy markers may serve as promising predictors of IM in HCC patients undergoing resectable LR.

## RESULTS

### Baseline demographic data

The clinical and biochemical features of the All Patient group and Cohorts 1 and 2 are shown in Table 1. The mean age was 63.1 ± 11.5 years, with a male predominance (73.1%). The etiology of HCC was Hepatitis B viral infection (HBV) (46.7%), HCV infection (28.4%), HBV and HCV co-infection (3.9%), and causes that were non-HBV/HCV-related (20.9%). Liver cirrhosis was present in one-third of the patients (32.3%). It was noted that 92 patients (17.2%) had a serum albumin level < 3.5 g/dl. A majority of the patients (77%) had undergone minor LR (≤ 2 segmentectomy). Among the 23% of patients who underwent major liver resection, 11.8% had 3-4 segmentectomy whereas 11.2% had > 4 segmentectomy. Thirty-nine patients (7.3%) had blood transfusions. According to the TNM stage and Barcelona Clinic Liver Cancer (BCLC) classifications, more patients were in stage I and II (83.6%) and stage 0 and A (63.9%), respectively. The expression of autophagy markers in the ANT tissues showed that more patient samples were stained positive for LC3 (59.8%) and negative for Beclin-1 (65.2%) and p62 (91.6%). The clinicopathological factors of the two cohorts were not significantly different.

**Table 1:** Basic demographic data of the All Patient group and Cohorts 1 and 2

Characteristics	All patients (n=535)	Cohort 1 (n=318)	Cohort 2 (n=217)	*p*-value
**Gender**				
*Female*	144 (26.9)	86 (27.0)	58 (26.7)	0.936
*Male*	391 (73.1)	232 (73.0)	159 (73.3)	
**Age (years)**	63.1±11.5	63.2±11.6	63.0±11.4	0.964
**HTN**	101 (18.9)	63 (19.8)	38 (17.5)	0.504
**DM**	59 (11.0)	36 (11.3)	23 (10.6)	0.794
**Alcohol**	129 (24.9)	78 (24.5)	51 (23.5)	0.785
**Smoking**	152 (28.4)	94 (29.6)	58 (26.7)	0.476
**HCC etiology**				
*Non HBVHCV*	112 (20.9)	68 (21.4)	44 (20.3)	0.926
*HBV*	250 (46.7)	150 (47.2)	100 (46.1)	
*HCV*	152 (28.4)	87 (27.4)	65 (30.0)	
*HBV+HCV*	21 (3.9)	13 (4.1)	8 (3.7)	
**Liver cirrhosis**				
*Negative*	362 (67.7)	216 (67.9)	146 (67.3)	0.876
*Positive*	173 (32.3)	102 (32.1)	71 (32.7)	
**AST (IU/L)**	55±38	55±38	54±39	0.881
**ALT (IU/L)**	50± 39	50±39	51±39	0.875
**Total bilirubin (mg/dl)**	0.79±0.34	0.78±0.34	0.81±0.35	0.417
**Albumin (g/dl)**				
*< 3.5*	92 (17.2)	54 (17.0)	38 (17.5)	0.873
*≥ 3.5*	443 (82.8)	264 (83.0)	179 (82.5)	
**Creatinine**	1.1±0.8	1.1±0.8	1.1±0.8	0.856
**Platelet count (x10^3^/ml)**	175±71	176±71	173±72	0.731
**INR**	1.07±0.10	1.08±0.13	1.08±0.14	0.620
**AFP (ng/dl)**	2797±13215	2963±14055	2555±11904	0.727
**ICG (%)**	8.3±5.3	8.4±5.4	8.2±5.2	0.759
**Child-Pugh score**				
*A*	484 (90.5)	289 (90.9)	195 (89.9)	0.694
*B*	51 (9.5)	29 (9.1)	22 (10.1)	
**Operative methods**				
*Minor LR*	412 (77.0)	244 (76.7)	168 (77.4)	0.852
*Major LR*	123 (23.0)	74 (23.3)	49 (22.6)	
**Operative margin (>1 cm)**				
*Negative*	150 (28.0)	87 (27.4)	63 (29.0)	0.672
*Positive*	385 (72.0)	231 (72.6)	154 (71.0)	
**Blood transfusions**	39 (7.3)	17 (5.3)	22 (10.1)	0.418
**Edmondson-Steiner grade**				
*I-II*	51 (9.5)	31 (9.7)	20 (9.2)	0.837
*III-IV*	484 (90.5)	287 (90.3)	197 (90.8)	
**Tumor number**				
*Single*	438 (81.9)	263 (82.7)	175 (80.6)	0.544
*Multiple*	97 (18.1)	55 (17.3)	42 (19.4)	
**Tumor size**				
*< 5 cm*	352 (65.8)	210 (66.0)	142 (65.4)	0.886
*≥ 5 cm*	183 (34.2)	108 (34.0)	75 (34.6)	
**TNM stage**				
*I-II*	447 (83.6)	268 (84.3)	179 (82.5)	0.693
*III-IV*	88 (16.4)	50 (15.7)	38 (17.5)	
**BCLC stage**				
*0-A*	342 (63.9)	205 (64.5)	137 (63.1)	0.584
*B-C*	193 (36.1)	113 (35.5)	80 (36.9)	
**LC3 staining non-tumor part**				
*Negative*	215 (40.2)	127 (39.9)	88 (40.6)	0.887
*Positive*	320 (59.8)	191 (60.1)	129 (59.4)	
**Beclin-1 staining non-tumor part**				
*Negative*	349 (65.2)	207 (65.1)	142 (65.4)	0.935
*Positive*	186 (34.8)	111 (34.9)	75 (34.6)	
**p62 staining non-tumor part**				
*Negative*	490 (91.6)	292 (91.8)	198 (91.2)	0.813
*Positive*	45 (8.4)	26 (8.2)	19 (8.8)	

Data shown as the mean ± standard deviation or number (%). HTN: hypertension; DM: diabetes mellitus; HBV: hepatitis B virus; HCV: hepatitis C virus; AST: aspartate aminotransferase; ALT: alanine aminotransferase; INR: international normalized ratio; AFP: alpha-fetoprotein; ICG: indocyanine green. Minor liver resection: ≤ 2 segmentectomy; major liver resection: ≥ 3 segmentectomy. BCLC stage: Barcelona Clinic Liver Cancer stage.

### Predictive factors related to immediate mortality in patients who had undergone liver resection

Factors associated with IM were analyzed in the All Patient group and in Cohorts 1 and 2 (Table 2). In the All Patient group, 27 patients (5.0%) had IM after surgical resection. Of these patients, 22 died of liver failure and 5 died of acute respiratory failure and sepsis related to liver failure. None of the patients had IM due to postoperative complications such as massive hemorrhage during or after surgery and cardiac arrest during surgery and anesthesia. Gender, age, pre-existing diseases (hypertension and diabetes mellitus), alcohol consumption and smoking habit, HCC etiology, presence of cirrhosis, Child-Pugh assessment of liver disease and tumor grade were not significantly different between patients with and without IM. By contrast, high serum ALT (98 ± 64 vs. 48 ± 35, *p*<0.0001), low serum albumin (< 3.5 g/dl; 13.0%, 12/92 vs. 3.4%, 15/443, *p*<0.0001) and major LR (11.4%, 14/123 vs. 3.2%, 13/412, *p*<0.0001) were significantly associated with IM. In the case of autophagy markers, IM was not associated with either Beclin-1 or p62 expression. However, a significantly higher proportion of patients negative for LC3 had IM (12.1%, 26/215 vs. 0.3%, 1/320, *p*<0.0001). When the patients from the two cohorts were analyzed separately, factors such as ALT levels, hypoalbuminemia, major LR, and LC3 remained significantly associated with IM in both groups.

**Table 2:** Basic factors associated with immediate mortality in the All Patient group and Cohorts 1 and 2

Immediate mortality	All patients		*p*-value	Cohort 1		*p*-value	Cohort 2		*p*-value
	Yes (n=27)	No (n=508)		Yes (n=16)	No (n=302)		Yes (n=11)	No (n=206)	
**Gender**									
*Female*	8 (29.6)	136 (26.8)	0.744	5 (31.3)	81 (26.8)	0.986	3 (27.3)	55 (26.7)	0.967
*Male*	19 (70.4)	372 (73.2)		11 (68.7)	221 (73.2)		8 (72.7)	151 (73.3)	
**Age (years)**	64.0±10.6	63.1±11.5	0.658	64.8±10.1	63.0±11.6	0.497	62.8±11.7	63.1±11.6	0.931
**HTN**	8 (29.6)	93 (18.3)	0.143	5 (31.3)	58 (19.2)	0.239	3 (27.3)	35 (17.0)	0.382
**DM**	2 (7.4)	57 (11.2)	0.538	1 (6.3)	35 (11.6)	0.511	1 (9.1)	22 (10.7)	0.868
**Alcohol**	6 (22.2)	123 (24.2)	0.814	3 (18.8)	75 (24.8)	0.581	3 (27.3)	48 (23.3)	0.762
**Smoking**	4 (14.8)	148 (29.1)	0.108	2 (12.5)	92 (30.5)	0.125	2 (18.2)	56 (27.2)	0.511
**HCC etiology**									
*Non HBVHCV*	7 (25.9)	105 (20.7)	0.235	5 (31.3)	63 (20.9)	0.375	2 (18.2)	42 (20.4)	0.623
*HBV*	16 (59.3)	234 (46.1)		9 (56.3)	141 (46.7)		7 (63.6)	93 (45.1)	
*HCV*	3 (11.1)	149 (29.4)		1 (6.2)	85 (28.1)		2 (18.2)	63 (30.6)	
*HBV+HCV*	1 (3.7)	20 (3.9)		1 (6.2)	13 (4.3)		0 (0)	8 (3.9)	
**Liver cirrhosis**									
*Negative*	19 (70.4)	343 (67.5)	0.758	11 (68.8)	205 (67.9)	0.942	8 (72.7)	138 (67.0)	0.693
*Positive*	8 (29.6)	165 (32.5)		5 (31.2)	97 (32.1)		3 (27.3)	68 (33.0)	
**AST (IU/L)**	82±49	53±36	0.062	80±48	53±36	0.080	86±52	54±37	0.048
**ALT (IU/L)**	98±64	48±35	**<.0001**	99± 65	48±35	**0.007**	96± 65	48±35	**0.037**
**Total bilirubin (mg/dl)**	0.77±0.31	0.80±0.34	0.754	0.79±0.31	0.78±0.34	0.953	0.76±0.32	0.81±0.35	0.588
**Albumin (g/dl)**									
*< 3.5*	12 (44.4)	80 (15.7)	**<.0001**	8 (50.0)	46 (15.2)	**<.001**	4 (36.4)	34 (16.5)	**<.001**
*≥ 3.5*	15 (55.6)	428 (84.3)		8 (50.0)	256 (84.8)		7 (63.6)	172 (83.5)	
**Creatinine**	1.3±1.2	1.0±0.8	0.327	1.2±1.1	1.0±0.7	0.585	1.4±1.4	1.0±0.8	0.423
**Platelet count (x10^3^/ml)**	176±43	175±72	0.954	174±42	176±72	0.880	177±48	174±73	0.808
**INR**	1.02±0.10	1.08±0.13	0.065	1.02±0.11	1.09±0.14	0.059	1.02±0.10	1.08±0.14	0.071
**AFP (ng/dl)**	2181±2466	2936±13548	0.101	2196±1498	3109±14408	0.070	2060±438	2683±12206	0.196
**ICG (%)**	8.6±4.9	8.3±5.3	0.759	8.6±5.1	8.3±5.4	0.888	8.6±4.9	8.1±5.2	0.768
**Child-Pugh score**									
*A*	26 (96.3)	456 (89.8)	0.083	15 (93.8)	274 (90.7)	0.194	11 (100)	184 (89.3)	0.253
*B*	1 (3.7)	52 (10.2)		1 (6.2)	28 (9.3)		0 (0)	22 (10.7)	
**Operative methods**									
*Minor LR*	13 (48.1)	399 (78.5)	**<.0001**	9 (56.3)	235 (77.8)	**0.048**	3 (27.3)	165 (80.1)	**<.0001**
*Major LR*	14 (51.9)	109 (21.5)		7 (43.7)	67 (22.2)		8 (72.7)	41 (19.9)	
**Operative margin (>1 cm)**									
*Negative*	5 (18.5)	145 (28.5)	0.258	2 (12.5)	85 (28.1)	0.171	3 (27.3)	60 (29.1)	0.895
*Positive*	22 (81.5)	363 (71.5)		14 (87.5)	217 (71.9)		8 (72.7)	146 (70.9)	
**Blood transfusions**	5 (18.5)	34 (6.7)	0.095	3 (18.7)	14 (4.6)	0.062	2 (18.2)	20 (9.7)	0.101
**Edmondson-Steiner grades**									
*I-II*	6 (22.2)	45 (8.9)	0.071	4 (25.0)	27 (8.9)	0.060	2 (18.2)	18 (8.7)	0.094
*III-IV*	21 (77.8)	463 (91.1)		12 (75.0)	275 (91.1)		9 (81.8)	188 (91.3)	
**Tumor number**									
*Single*	21 (77.8)	417 (82.1)	0.571	13 (81.2)	250 (82.8)	0.875	8 (72.7)	167 (81.1)	0.495
*Multiple*	6 (22.2)	91 (17.9)		3 (18.8)	52 (17.2)		3 (27.3)	39 (18.9)	
**Tumor size**									
*< 5 cm*	19 (70.4)	333 (65.6)	0.607	11 (68.8)	199 (65.9)	0.814	8 (72.7)	134 (65.0)	0.602
*≥ 5 cm*	8 (29.6)	175 (34.4)		5 (31.2)	103 (34.1)		3 (27.3)	72 (35.0)	
**TNM stage**									
*I-II*	22 (81.5)	425 (83.7)	0.766	13 (81.2)	255 (84.4)	0.733	9 (81.8)	170 (82.5)	0.952
*III-IV*	5 (18.5)	83 (16.3)		3 (18.8)	47 (15.6)		2 (18.2)	36 (17.5)	
**BCLC stage**									
*0-A*	15 (55.6)	327 (64.4)	0.353	9 (56.2)	196 (64.9)	0.481	6 (54.5)	131 (63.6)	0.545
*B-C*	12 (44.4)	181 (35.6)		7 (43.8)	106 (35.1)		5 (45.5)	75 (36.4)	
**LC3 staining non-tumor part**									
*Negative*	26 (96.3)	189 (37.2)	**<.0001**	16 (100)	111 (36.8)	**<.0001**	10 (90.9)	78 (37.9)	**<.0001**
*Positive*	1 (3.7)	319 (62.8)		0 (0)	191 (63.2)		1 (9.1)	128 (62.1)	
**Beclin-1 staining non-tumor part**									
*Negative*	21 (77.8)	328 (64.6)	0.160	11 (68.8)	196 (64.9)	0.753	10 (90.9)	132 (64.1)	0.068
*Positive*	6 (22.2)	180 (35.4)		5 (31.2)	106 (35.1)		1 (9.1)	74 (35.9)	
**p62 staining non-tumor part**									
*Negative*	25 (92.6)	465 (91.5)	0.847	14 (87.5)	278 (92.1)	0.517	11 (100)	187 (90.8)	0.292
*Positive*	2 (7.4)	43 (8.5)		2 (12.5)	24 (7.9)		0 (0)	19 (9.2)	

Immediate mortality is defined as mortality within 3 months after resection. Data shown as mean ± standard deviation or number (%). HTN: hypertension; DM: diabetes mellitus; HBV: hepatitis B virus; HCV: hepatitis C virus; AST: aspartate aminotransferase; ALT: alanine aminotransferase; INR: international normalized ratio; AFP: alpha-fetoprotein; ICG: indocyanine green. Minor liver resection: ≤ 2 segmentectomy; major liver resection: ≥ 3 segmentectomy. BCLC stage: Barcelona clinic liver cancer.

In multivariate analysis, the Cox proportional hazard model identified that patients with an absence of LC3 in the ANT tissues had the highest risk of IM (Hazard ratio [HR] 40.8; 95% confidence interval [CI]: 5.14-325; *p*<0.0001) followed by those with serum albumin level below 3.5 g/dl (HR 2.88; 95% CI: 1.11-7.52; *p*=0.03) (Table 3).

**Table 3:** Multivariate analyses for immediate mortality in all the HCC patients who had undergone liver resection

Variable	Hazard ratio	95% CI	*p*-value
**LC3 staining of non-tumor part**			
*Positive*	1		
*Negative*	40.8	5.14-325	**<.0001**
**Serum albumin level (g/dl)**			
*≥ 3.5*	1		
*< 3.5*	2.88	1.11-7.52	**0.030**
**Serum ALT level (IU/L)**	0.98	0.98-1.01	0.058
**Operative methods**			
*Minor LR*	1		
*Major LR*	0.53	0.21-1.39	0.200

CI: confidence interval; ALT: alanine aminotransferase; minor liver resection: ≤ 2 segmentectomy; LR: liver resection; major liver resection: ≥ 3 segmentectomy.

#### Prognosis of immediate mortality defined by LC3 expression in the adjacent non-tumor tissues and serum albumin level

**Figure 1:**
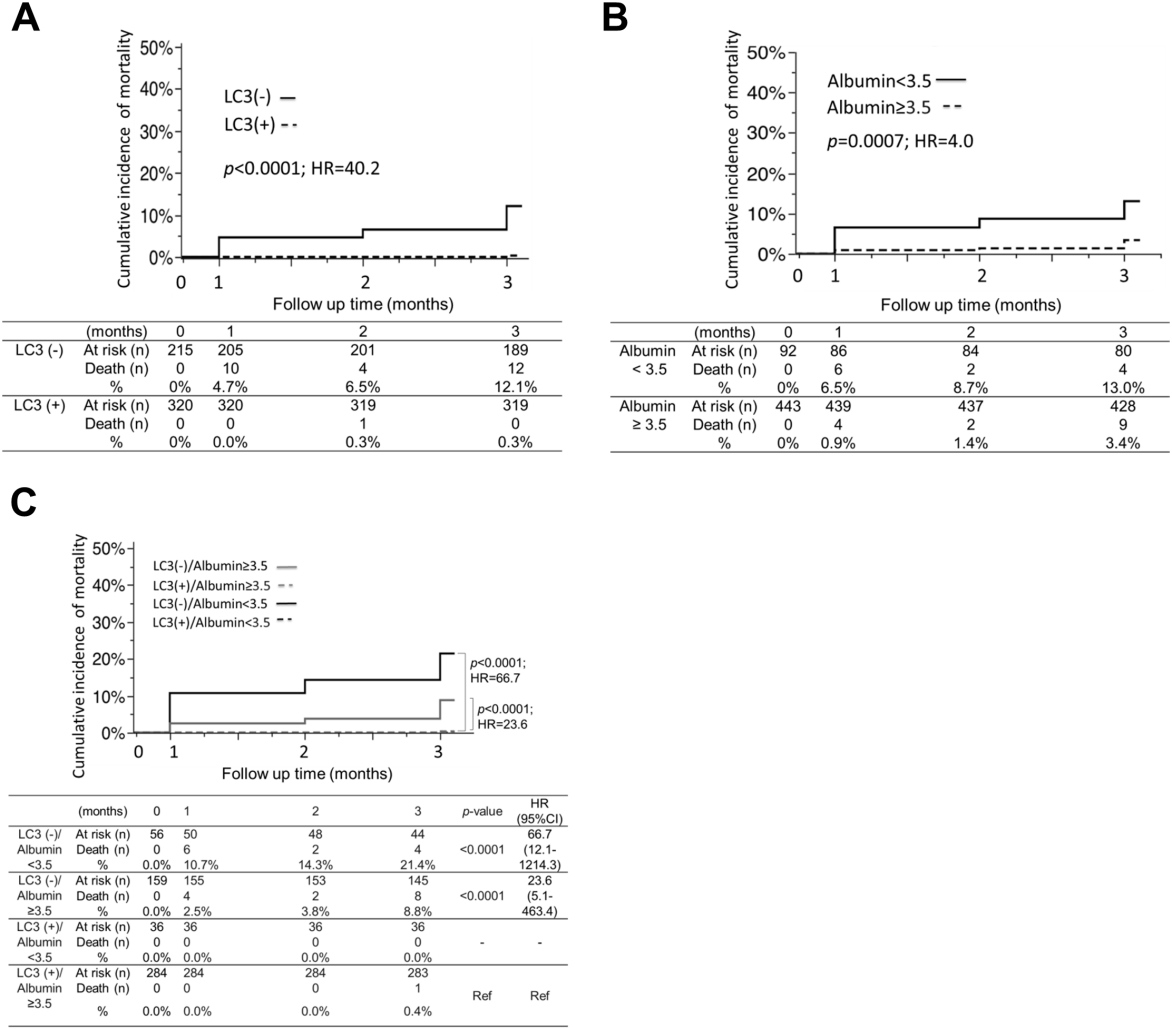
The cumulative incidence of post-hepatectomy immediate mortality associated with the expression of LC3 in the adjacent non-tumor tissues and/or serum albumin level Based on Kaplan-Meier analyses, HCC patients with **(A)** the absence of LC3, **(B)** hypoalbuminemia (< 3.5 g/dl) and **(C)** both the absence of LC3 and the presence of hypoalbuminemia had significantly higher risk of immediate mortality.

Given that LC3 expression and serum albumin level are factors associated with IM, Kaplan-Meier survival analyses were performed to investigate the prognosis of IM after LR. The absence of LC3 was significantly correlated with an increase in IM (HR 40.2; 95% CI: 8.6-718.6; *p*<0.0001; Figure 1A). The 3-month cumulative incidence of mortality was 0.3% and 12.1% in the presence and absence of LC3, respectively. Similarly, low serum albumin level (< 3.5 g/dl) was also significantly correlated with IM (HR 4.0; 95% CI: 1.9-8.6; *p*=0.0007; Figure 1B). The 3-month cumulative incidence of mortality was 3.4% and 13.0% in patients with serum albumin ≥ 3.5 g/dl and < 3.5 g/dl, respectively. Furthermore, both the absence of LC3 and hypoalbuminemia were also significantly correlated with IM (HR 66.7; 95% CI: 12.1-1214.3; *p*<0.0001; Figure 1C). The 3-month cumulative incidence of mortality was 21.4% in patients with both the absence of LC3 and hypoalbuminemia, compared to only 0.4% among those with both the presence of LC3 and serum albumin levels ≥ 3.5 g/dl. Moreover, having both an absence of LC3 and albumin ≥ 3.5 g/dl was significantly correlated with IM (HR 23.6; 95% CI: 5.1-463.4; *p*<0.0001; Figure 1C), and the 3-month cumulative incidence of mortality was 8.8%.

## DISCUSSION

With great advances in the methodological detection of HCC, perioperative management, and operative techniques, LR has become one of the standard management methods for resectable HCC [4–6, 28]. However, major complications that have arisen from LR, in particular, PHLF, have accounted for up to 10% of mortality rates [10, 11]. The early identification of predictor(s) associated with PHLF, preceding clinical evidence of post-surgical complications, may aid in identifying patients at risk of IM so that supportive and prophylactic therapies can be given as early as possible to enhance patient survival. In the current study, a total of 535 HCC patients who had undergone LR at two local hospitals were examined. Our results revealed that patients positive for LC3 in the ANT tissues and with high serum albumin levels (≥ 3.5 g/dl) are significantly less likely to develop post-hepatectomy IM. These data suggest that patients lacking LC3 and/or having hypoalbuminemia are more prone to IM during the first three months of the post-hepatectomy recovery period.

The involvement of autophagy in the field of hepatology has been widely studied, and a perturbation in autophagy function in the liver has been reported to have an effect on both the physiology and disease development of the liver [29, 30]. HCC patients have been reported to exhibit a decrease in autophagy function, and the use of autophagy-promoting therapeutic drugs has been demonstrated to have a beneficial effect in minimizing liver injury [31, 32]. To date, the prognostic significance of LC3 in predicting both tumor recurrence and the overall survival of HCC patients has been reported [22, 33]. However, the association between LC3 and IM has not been investigated. This is the first report that demonstrates the feasibility of using LC3 as a predictive factor of IM in HCC patients who have undergone LR, independent of the well-established clinicopathologic stages and results. Therefore, LC3, with a high HR of 40.8, may serve as a strong biomarker in predicting IM in patients undergoing curative resection. The inclusion of IHC examination of the ANT tissues for LC3 expression during LR to evaluate its effect in inducing autophagy and regenerative capacity could provide important information for critical post-hepatectomy monitoring and supportive therapy.

Using a mouse model, we have previously shown that autophagy is induced after LR, and its induction promotes the growth and regeneration of the liver, accompanied by a reduction in liver injury [27]. Liver regeneration and hepatocyte proliferation are crucial in restoring both the normal hepatic mass and functional capacity of the future remnant liver, and liver weight is normally restored by postoperative day (POD) 8–15, followed by lobular reorganization [34]. In this study, the decrease in the IM rate observed in patients with high LC3 expression clearly demonstrates that autophagy is likely to have an impact on post-hepatectomy IM.

Albumin is a major blood protein biosynthesized by the liver, and its production is affected by factors including hormonal and environmental changes, nutritional status, toxin exposure, and trauma stress [35]. Patients with liver or kidney disease, malnutrition, or a low-protein diet may exhibit clinical hypoalbuminemia. In addition, serum albumin is essential for the maintenance of colloid osmotic pressure during surgery, and a drop in albumin level post-operatively is an indicator of trauma degree [36, 37]. In patients with LR, albumin biosynthesis by the liver may become compromised as a result of surgical trauma or the loss or decomposition of the liver. Resectable HCC patients with pre-operative hypoalbuminemia are therefore at higher risk of post-operative complications, and albumin is often supplemented prophylactically prior to surgery [36, 38–40]. These observations support our findings that patients with hypoalbuminemia are more susceptible to IM and the measurement of serum albumin prior to surgery may serve as a good biomarker in predicting post-hepatectomy IM.

Pre-operative risk assessments, such as computed tomography-based volumetric analysis to predict the volume of the remnant liver [41] and ICG-R15 to estimate preoperative hepatic functional reserve [2], are often employed to identify patients at risk of PHLF and to assess appropriate operative methods. However, PHLF remains one predominant cause of morbidity and mortality and is manifested by the progressive malfunctioning of multiple organs [7, 42]. According to the International Study Group of Liver Surgery (ISGLS), PHLF is defined as a post-surgical acquired impairment of the liver to maintain its synthetic, excretory, and detoxifying function, which is characterized by an increase in INR and concomitant hyperbilirubinemia on or after POD5 [8]. In the ‘50-50 criteria’ system, the pro-thrombin time (PT) and serum bilirubin (SB) of patients who have undergone LR are measured on PODs 1, 3, 5 and 7. Patients with PT < 50% and SB > 50 μMol/l on POD5 carry a 59% risk of developing early PHLF [12] and may be subjected to aggressive laboratory investigation for complications associated with PHLF. In this study, one major advantage of using serum albumin and LC3 as predictive markers of IM is that serum albumin level before LR can be routinely measured with ease, and IHC staining of ANT tissues retrieved from LR for LC3 expression can be performed immediately. Given that both laboratory results are obtainable at a much earlier time, patients identified at risk may be given more critical post-operative monitoring and care for possible clinical complications and hepatic failure.

The limitations of the current study include the following: this retrospective study may result in an unintended bias. Although patients lacking LC3 in the ANT tissues could predict post-hepatectomy IM, these studies were performed in the East Asian region. More studies are required to validate such findings in Western regions with different ethnicity. LC3 is a hallmark of autophagy, and when autophagy is inactivated and activated, LC3 exists as soluble LC3-I and lipidated LC3-II forms, respectively. One means of measuring autophagy activity is through the determination of autophagic flux – detection of LC3-II accumulation in the presence of lysosomal degradation inhibitor via Western blot analysis. In our study, the use of IHC staining for LC3 expression analysis could not distinguish between LC3-I and LC3-II, and therefore, the presence of LC3 expression is not reflectively of autophagic activity. Hence, our findings are not applicable for therapeutic use and should be considered within the context of these limitations. In addition, this study does not include PT and SB measurements on POD5 and therefore, the association between mortality rate of PHLF and ‘50-50 criteria’ could not be established.

In summary, our study revealed that the absence of LC3 expression—a biomarker of autophagy—in the ANT tissues and hypoalbuminemia—a sign of poor reserve liver function—were strongly associated with a high risk of IM in HCC patients who had undergone LR. Furthermore, both the absence of LC3 and hypoalbuminemia were significantly associated with increased IM. The staining of ANT tissues for LC3 expression and serum albumin could potentially serve as prognostic factors to identify patients at risk of post-operative IM after surgical resection for HCC.

## MATERIALS AND METHODS

### Patients

This retrospective study enrolled 318 HCC patients who had undergone surgical resection between 2010 and 2014 at E-Da Hospital, I-Shou University, Kaohsiung, Southern Taiwan (Cohort 1) and 217 HCC patients who had undergone surgical resection between 2010 and 2013 at Chunghua Christian Hospital, Chunghua, Central Taiwan (Cohort 2). All patients were diagnosed with HCC using histopathology examination performed by two independent pathologists. All patients had regular post-operation follow-up every month for 3 months. Immediate mortality (IM) was defined as patients who had died within three months after surgical resection.

The demographic, clinical, and pathological data related to this study were collected and analyzed (n=535). Clinicopathological parameters, including demographic data, hepatitis markers, biochemistry analyses, operative methods, and tumor size, were examined. ICG-R15 for pre-operative risk assessment was also conducted on these patients [2]. The stages of HCC were established using the American Joint Committee on Cancer TNM staging system and BCLC staging system [43, 44]. The functional status of the liver was evaluated using the Child-Pugh scoring system [45]. Tumor histological grading was performed using the Edmondson–Steiner system [46]. Major LR was defined as the surgical removal of more than 2 Couinaud's segments. The ANT tissues that were between 0.5 – 5 cm from the negative operative margin was collected and stored in 4% paraformaldehyde until required. The collection of the human specimens in this study was approved by the institutional review board at E-Da Hospital, I-Shou University, and Chunghua Christian Hospital. Written informed consent was obtained from all patients before enrollment.

### Immunohistochemical staining and scoring

The formalin-fixed, paraffin embedded tissue samples were assembled to build tissue microarray blocks using a commercially available manual tissue microarray (Array Biotechnology Co., Taiwan). The horseradish peroxidase/diaminobenzidine detection system was used on 4-μm tissue sections for immunohistochemistry (IHC) staining according to the manufacturer's instructions with minor modifications. The primary antibodies used in the investigation were for LC3 (1:200, NB100-2220, Novus Biologicals, Littleton, CO, USA), Beclin-1 (1:100, ab51031, Abcam, Cambridge, UK), and p62 (1:100, H00008878-M01, Abnova, Taipei, Taiwan).

**Figure 2:**
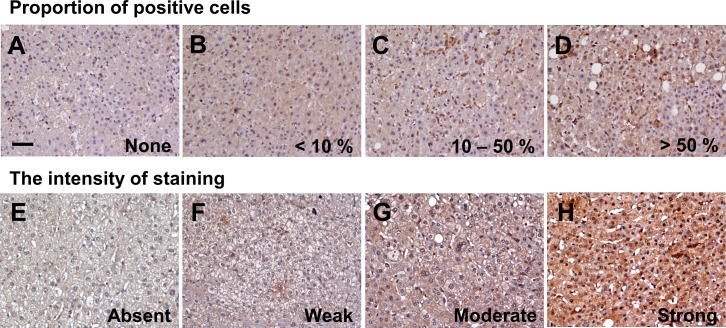
LC3 expression in the adjacent non-tumor tissues by immunohistochemistry staining Representative images of areas according to the proportion of positive cells **(A–D)** and intensity of staining **(E–H)**. (A) none, (B) < 10%, (C) 10–50%, (D) > 50%; and staining (E) absent, (F) weak, (G) moderate, (H) strong. (upper panel, x200; lower panel, x400).

The expression of autophagy-related proteins (LC3, Beclin-1, and p62) in the ANT tissues was evaluated using the semi-quantitative immunoreactive score (IRS) method, according to the guidelines previously reported [47]. The immunostaining scores were calculated according to the intensity and percentage of positive staining of all slides in this study. The intensity score was defined as 0 (no staining), 1 (weak staining), 2 (moderate staining) and 3 (strong staining). The percentage score was defined as 0 (None, no staining), 1 (< 10% positivity), 2 (10–50%) and 3 (≥ 50%) (Figure 2, Supplementary Figures 1 and 2). The intensity score and percentage score were multiplied together to obtain a total score. The total scores were further assigned, and samples with scores 0-1 were defined as negative, while those with scores 2 and above were defined as positive. All the slides were evaluated independently by two investigators (Lin CW and Koh KW) in a blinded manner whereby clinicopathological information and clinical outcomes were not revealed. Cases with discrepancies in the score were discussed together with other pathologists until consensus was reached to confirm the IRS score.

### Data analysis and statistics

Data management and statistical analyses were performed using SPSS ver. 18.0 (SPSS, Chicago, IL, USA). Associations between categorical variables and operative mortality were evaluated using Pearson's χ2 test or Fisher's exact test, as appropriate. Group means (presented as the mean ± standard deviation) were compared using analysis of variance and Student's t-test, where appropriate. To evaluate whether the variables selected in the univariate analysis were independent risk factors of operative mortality, multivariate analyses were evaluated using Cox's proportional hazard regression analysis. Kaplan–Meier analysis and the log-rank test were performed by comparing the differences in the cumulative incidence of operative mortality between determinants. All statistical analyses were based on a two-sided hypothesis tests with a significance level of *p*-value < 0.05.

## SUPPLEMENTARY MATERIALS FIGURES



## Abbreviations

ANT: Adjacent non-tumor
AASLD: AmericanAssociation for the Study of Liver Diseaes
AFP: alpha-fetoprotein
ALT: alanine aminotransferase
BCLC: Barcelona Clinic liver cancer
C–P: child–pugh
CI: confidence interval
HR: hazard ratio
HCC: hepatocellular carcinoma
IM: immediate mortality
IRS: immuno-reactive-score
ICG-R15: indocyanine green retention rate
LR: liver resection
