# Supplementary material for: The autophagy marker LC3 strongly predicts immediate mortality after surgical resection for hepatocellular carcinoma
Source: Oncotarget. 2017 Aug 1;8(54):91902–13. doi: 10.18632/oncotarget.19763 (PMC5696150; doi:10.18632/oncotarget.19763)
Supplement: Supplementary file 1 [file oncotarget-08-91902-s001.pdf]

The autophagy marker LC3 strongly predicts immediate mortality after surgical resection for hepatocellular carcinoma

SUPPLEMENTARY MATERIALS

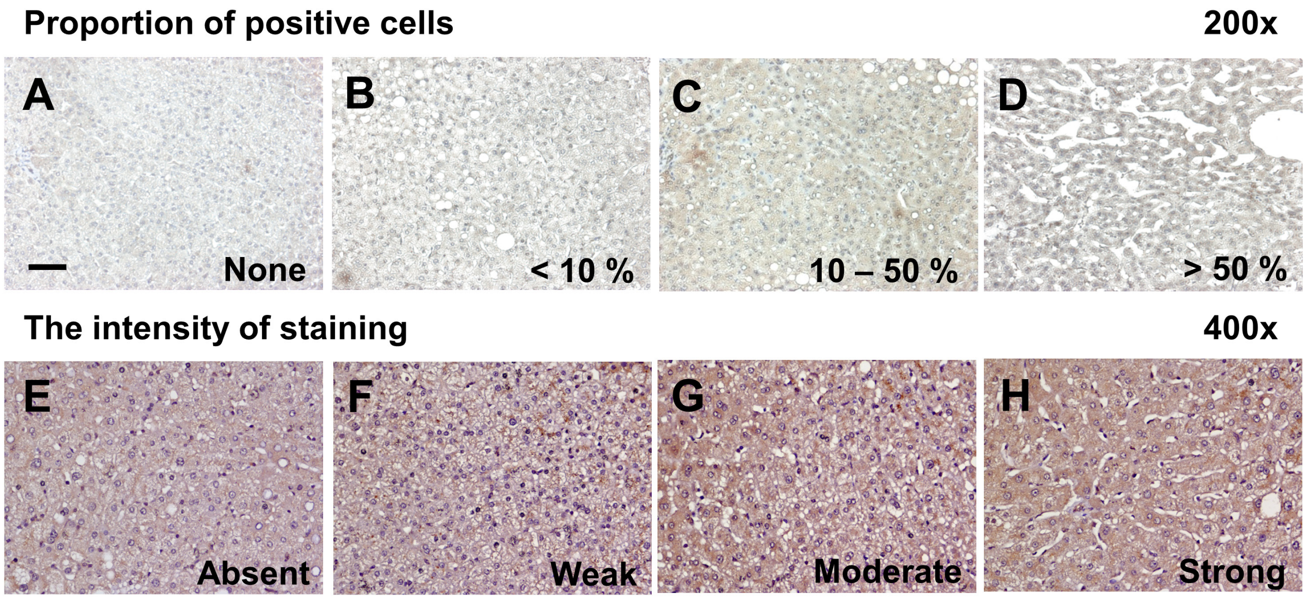

Supplementary Figure 1: Beclin-1 expression in the adjacent non-tumor tissues by immunohistochemistry staining. Representative images of areas according to the proportion of positive cells **(A–D)** and intensity of staining **(E–H)**. (A) none, (B) < 10%, (C) 10–50%, (D) > 50%; and staining (E) absent, (F) weak, (G) moderate, (H) strong. (upper panel, x200; lower panel, x400).

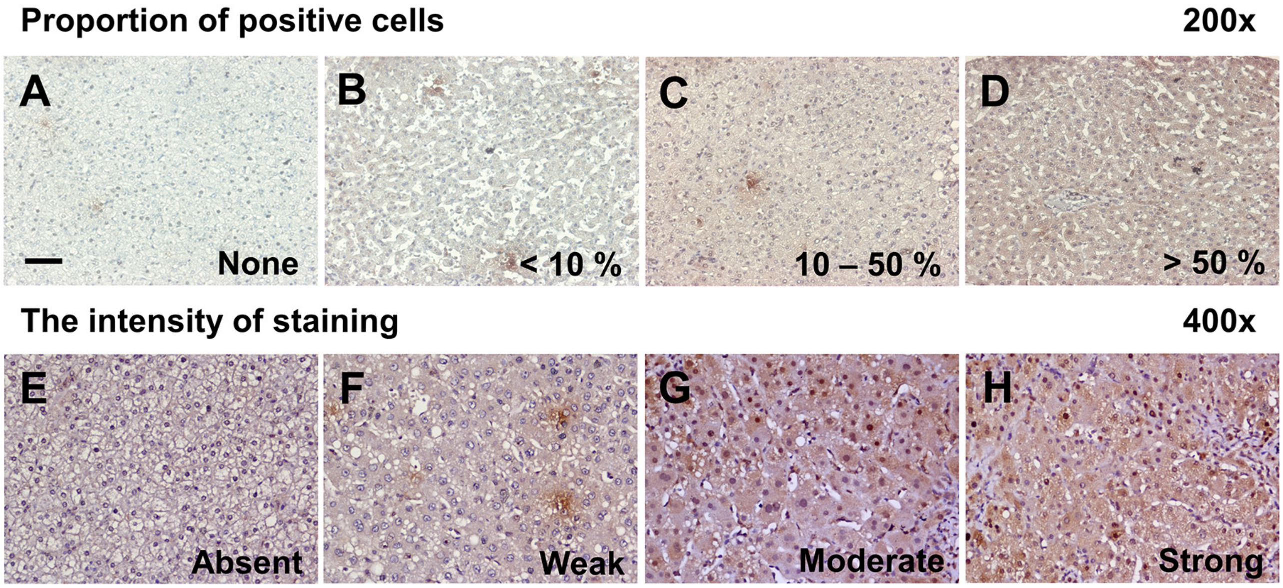

Supplementary Figure 2: p62 expression in the adjacent non-tumor tissues by immunohistochemistry staining. Representative images of areas according to the proportion of positive cells **(A–D)** and intensity of staining **(E–H)**. (A) none, (B) < 10%, (C) 10–50%, (D) > 50%; and staining (E) absent, (F) weak, (G) moderate, (H) strong. (upper panel, x200; lower panel, x400).
